# Promoter Binding and Nuclear Retention Features of Zebrafish IRF Family Members in IFN Response

**DOI:** 10.3389/fimmu.2022.861262

**Published:** 2022-04-06

**Authors:** Li-Li An, Xiang Zhao, Xiu-Ying Gong, Yi-Lin Li, Zi-Ling Qu, Hao-Yu Sun, Wen-Hao Guo, Cheng Dan, Jian-Fang Gui, Yi-Bing Zhang

**Affiliations:** ^1^State Key Laboratory of Freshwater Ecology and Biotechnology, Institute of Hydrobiology, Chinese Academy of Sciences, Wuhan, China; ^2^College of Advanced Agricultural Sciences, University of Chinese Academy of Sciences, Beijing, China; ^3^The Innovation Academy of Seed Design, Chinese Academy of Sciences, Wuhan, China; ^4^Key Laboratory of Aquaculture Disease Control of Ministry of Agriculture, Institute of Hydrobiology, Chinese Academy of Sciences, Wuhan, China

**Keywords:** interferon regulatory factor, DNA binding, nuclear import, interferon expression, nuclear localization signal

## Abstract

Interferon regulatory factors (IRFs) constitute a family of transcription factors that synchronize interferon (IFN) antiviral response through translocating to nucleus and binding to the promoters of IFN and IFN-stimulated genes (ISGs). Fish contain 11 IRF members; however, whether or how fish IRF family genes function in IFN response remains limited. Herein, we determine the regulatory roles of 11 zebrafish IRF family members in IFN response relevant to their subcellular localization and promoter binding. Zebrafish IRF family members display three patterns of constitutive localization, only in nucleus (IRF1/2/9/11), only in cytoplasm (IRF3/5/7), and largely in nucleus with small amounts in cytoplasm (IRF4b/6/8/10). DNA pull-down assays confirm that all zebrafish IRF proteins are capable to bind fish IFN promoters, albeit to various degrees, thus regulating IFN gene transcription as activators (IRF1/3/5/6/7/8/9/11) or repressors (IRF2/4b/10). Further characterization of distinct IFN gene activation reveals that IRF1/3/5/6/7/8/9/11 efficiently stimulate zebrafish IFNφ1 expression, and IRF1/7/11 are responsible for zebrafish IFNφ3 expression. Two conserved basic residues within the helix α3 of DNA binding domains (DBDs) contribute to constitutive or inducible nuclear import for all zebrafish IRF family members and DNA binding for most members, thereby enabling them to function as transcription factors. Our results reveal a conserved and general mechanism that specifies zebrafish IRF family proteins to nuclear import and DNA binding, thereby regulating fish IFN response.

## Introduction

A hallmark feature of innate antiviral immunity is the production of interferons (IFNs) and downstream IFN-stimulated genes (ISGs) (1). In virus-infected cells, cellular pattern recognition receptors (PRRs), such as retinoic acid-inducible gene I (RIG-I)-like receptors (RLRs), Toll-like receptors (TLRs) and cyclic GMP-AMP Synthase (cGAS), rapidly recognize virally-derived nucleic acids, finally converging on the activation of transcription factors of IFN regulatory factor (IRF) family to initiate IFN and ISG transcription (1). Since IRF1 is defined as the funding member of IRF family (2), 9 IRF proteins have been identified in mammals. Structurally, they all bear a well-conserved N-terminal DNA-binding domain (DBD) of ∼120 amino acids, and a less conserved IRF-association domain (IAD)1 or IAD2 (3, 4). The conserved DBD forms a helix-turn-helix structure required for recognizing and occupying an IRF-binding element (IRF-E)/IFN-stimulated response element (ISRE) in the promoters of IFNs and ISGs (3, 5). The IAD1/IAD2 is responsible for the association of different transcription factors to shape IFN gene transcription (4).

Genetic data have revealed the relevance of IRF family members to IFN responses (6–10). IRF3 and IRF7 are two primary regulators of IFN expression downstream of signaling pathways triggered by TLRs (such as TLR3/7/9), RLRs and DNA sensors (such as cGAS) (1). IRF9 is essential for IFN signaling and host antiviral state as a component of ISGF3 (11). Although IRF1/2/4/5/8 are mainly reported to regulate immune cell development, differentiation and phonotype (6, 12–14), there is definite evidence that they are involved in host IFN response. IRF1 and IRF2 are originally characterized as two antagonistic regulators of IFN system, likely through competitive binding to IFN gene promoters (15). IRF5 is an IFNα activator when present as homodimers or heterodimers with IRF3, and instead, is a repressor when interacting with and inhibiting IRF7 to bind IFN gene promoters (9, 16, 17). IRF8 activates IFN response, together with IRF3/7 in DCs (18), and with IRF3 in human blood monocytes (7). While IRF4 competes with IRF5 to downregulate the expression of proinflammatory cytokines rather than IFNs in macrophages (19), it still displays a weak binding affinity to ISRE/IRF-E motifs (12). Recent studies have linked IRF6 to IFNβ-induced liver injury (20) and its inhibitory role in poly(I:C)-triggered IFN promoter activation.

IRF proteins function in nucleus, but with diversified subcellular localization in resting cells. IRF3/5/7 constitutively reside in cytoplasm and following virus infection, they are translocated to nucleus for IFN transcription (9, 21–23). On the contrary, IRF1/2/9 reside in nucleus (24–26); IRF4/8 are mainly expressed in nucleus and partially in cytoplasm (22). Nuclear import of IRF proteins is driven by nuclear localization signal (NLS), which is often composed of one (monopartite), two (bipartite) or three (tripartite) stretches rich of lysines (K) and arginines (R) (27, 28). However, available data suggest that the NLS sequences of IRF family members are not strictly conserved (22, 29).

Besides IRF1-9 mentioned above, fish have IRF10 and IRF11 that are lost in mammals (28, 30). Functionally, fish IRF1/3/7/11 act as activators of IFN response (28, 31–37) and IRF2/10 as repressors (38, 39). Interestingly, IRF2 selectively upregulates certain fish IFN expression (40), and fish IFN induces itself expression *via* IRF9 (41). Upon infection, IRF3/7 undergo a cytoplasmic to nuclear translocation and subsequent binding to fish IFN gene promoters (31, 35). Like mammalian counterparts, fish IRF1/9 constitutively reside in nucleus (34, 41, 42). However, overexpression of fish IRF8 obtains inconsistent protein localization and function in different fish species (43, 44). Similar phenomenon happens to mammals. For example, human IRF3’NLS is recently believed as a bipartite NLS (29) rather than a monopartite NLS identified previously (21), highlighting that the mechanisms specifying IRFs to nucleus are largely unknown.

Recently we showed that two basic residues (K78, R82) within DBD helix α3 of zebrafish IRF11 has an integrated function of nuclear import and DNA binding (28). In the present study, we found that that both residues are conserved across zebrafish IRF family members, and mutation of both resulted in a complete function loss of zebrafish IRF family members in regulating IFN expression. Mechanistically, both residues were required for nuclear import of all 11 zebrafish IRF family members and the promoter binding of most members, thus enabling IRF proteins to act as activators of fish IFN response (IRF1/3/5/7/8/9/11) or as repressors (IRF2/4/10).

## Materials and Methods

### Cells, Virus and Zebrafish

Epithelioma papulosum cyprini (EPC) cells and grass carp (*Ctenopharyngodon idellus*) ovary (CO) cells were cultured in medium 199 basic (Gibco) supplemented with 10% fetal bovine serum (FBS) at 28°C, and HEK293T cells were in DMEM basic (Gibco) with 10% FBS at 37°C in a humidified incubator containing 5% CO_2_. Spring viraemia of carp virus (SVCV) and grass carp reovirus (GCRV) were propagated in EPC cells and CO cells, respectively, and the titer was determined with a 50% tissue culture infective dose (TCID_50_) assay according to Reed and Much methods. Zebrafish (*Danio rerio*) strain AB were raised according to standard protocols, which was approved by the Animal Care and Use Committee of Institute of Hydrobiology, Chinese Academy of Sciences. For viral infection, zebrafish adults (2-month-old) were injected i.p. (intraperitoneally injection) with SVCV.

### Plasmids

The open reading frames (ORF) of 11 zebrafish IRF family genes (IRF1: NM_205747.1; IRF2: NM_001328374; IRF3: NM_001143904; IRF4b: XM_021468630; IRF5: NM_001327817; IRF6: NM_200598; IRF7: NM_200677; IRF8: NM_001002622; IRF9: NM_205710; IRF10: NM_212879; IRF11: NM_001040352) were inserted into the *Nhe* I/*Kpn* I site of pEGFP-N3 to construct expression plasmids expressing respective IRF protein that was fused to GFP (IRF1/2/3/4b/5/6/7/8/9/10/11-wt). 11 zebrafish IRF mutants, including IRF1-mut (K78A/R82A), IRF2-mut (K77A/R81A), IRF3-mut (K76A/R80A), IRF4b-mut (K88A/R92A), IRF5-mut (K80A/R84A), IRF6-mut (K80A/R84A), IRF7-mut (K75A/R79A), IRF8-mut (K77A/R81A), IRF9-mut (K81A/R85A), IRF10-mut (K80A/R84A) and IRF11-mut (K78A/R82A), were obtained by mutation of the indicated two residues to alanine based on the wild type plasmids. Promoter-driven luciferase constructs including CaIFNpro-luc, DrIFNφ1pro-luc, DrIFNφ3pro-luc, and ISRE-luc, were described previously (31, 32, 34).

### Transfection, Luciferase Activity Assays

Plasmid transfection was carried out by Polyethylenimine Linear (PEI, MW25000, Aldrich, USA) according to the manufacturer’s protocol or our previous reports (31, 36, 45). Luciferase assays were performed as described previously (31, 36). Briefly, EPC cells seeded in 48-well plates overnight were transfected with various plasmids at a ratio of 1:10:10 (pRL-TK, promoter-driven luciferase reporter plasmid, expression construct). If needed, the cells were simultaneously transfected with poly(I:C). 30h later, the cells were collected for detecting luciferase activities in a Junior LB 9509 luminometer (Berthold, Pforzheim, Germany) using Dual-Luciferase Reporter Assay System (Promega). The relative expression values of luciferase activities were normalized to the amounts of Renilla luciferase activities according to the protocol. The results were the representative of at least three independent experiments, each performed in triplicate.

### RNA Extraction, cDNA Synthesis, and Real Time-PCR

SVCV-injected zebrafish were sampled for different tissues, followed by extraction of total RNAs using TRIZOL Reagent (AIDLAB, China). First-strand cDNA was synthesized using random primers (AIDLAB, China). RT-qPCR was performed with Universal Blue qPCR SYBR Green Master Mmix (YEASEN, China) in a DNA Engine Chromo 4 real-time system (BioRad, USA). Gene expression values were normalized to β-actin in a same sample. The results were the representative of at least three independent experiments, each performed in triplicate. The primers used in this study were listed in **Table 1**.

**Table 1:** Primers used in this study.

Primer names	Sequence (5’-3’)	Primer names	Sequence (5’-3’)
DrIRF1-RT-F	AGATGCCTGTCTGTTCAAGC	DrIRF8-RT-R	TTGTCCACCATCTGACCTCCG
DrIRF1-RT-R	ATGCGGTAAACTCTCACC	DrIRF9-RT-F	ACACGCCTTCGATGTGC
DrIRF2-RT-F	TTCCAGATTCCGTGGATGC	DrIRF9-RT-R	ATGTTGATGGCCTGTGGTG
DrIRF2-RT-R	TTCACCTCCTCGATGTCG	DrIRF10-RT-F	ACTACAGACAGAACCAGG
DrIRF3-RT-F	AACAGCGACGATGTGCTC	DrIRF10-RT-R	TGCATGGACAGGTGTG
DrIRF3-RT-R	ATCTGGGTTCCTGGATCC	DrIRF11-RT-F	AGGACGCAACACTGTTCAGG
DrIRF4b-RT-F	TTCGGATTCCGTGGAAGCACG	DrIRF11-RT-R	TGTGAATCTCTCCAGGGAG
DrIRF4b-RT-R	TTGGATAATTGGGAGAGCC	DrIFNφ1-F	ACGACAGAATCTCTGAACCT
DrIRF5-RT-F	TACCCAGGACTGCATTGGC	DrIFNφ1-R	GTCAGGACTAAAAACTTCAC
DrIRF5-RT-R	TTGACTGACTGATCGCACAC	DrIFNφ3-F	TTCTGCTTTGTGCAGGTTTG
DrIRF6-RT-F	AAGTATCAGGAAGGAGTGG	DrIFNφ3-R	GGTATAGAAACGCGGTCGTC
DrIRF6-RT-R	TTCAGGAGTGTCTGGGATG	DrMxb-F	CTGGAGCAGGTGTTGGTATC
DrIRF7-RT-F	TCAGTTTGGACCGTGGCTC	DrMxb-R	ATGCCTAAAGTCCTTTCGCC
DrIRF7-RT-R	TGAGGACGAATGATGCG	DrActin-F	CACTGTGCCCATCTACGAG
DrIRF8-RT-F	AGGTAAAGGCACAGTCACC	DrActin-R	CCATCTCCTGCTCGAAGTC

### Subcellular Localization Assays

Subcellular localization was determined in HEK293T cells by GFP reporter assays (31). Briefly, HEK293T cells plated overnight on microscopic cover glasses in six-well plates were transfected with plasmids expressing respective zebrafish IRF fused to GFP. 24 h later, the transfected cells were washed three times with PBS, fixed with 4% (v/v) paraformaldehyde at room temperature for 30 min, washed again with PBS three times, incubated with 0.2% Triton X-100 for 15 min, and finally stained with DAPI (500µg/ml) for 30 min. Cells were examined by a confocal microscope [ZEN Blue Lite confocal system. Objectives: ×40; analysis software: ZEN 2.3 (blue edition)].

Subcellular localization of the endogenous IRF3 and IRF7 protein in CO cells was determined by immunofluorescence analyses. Briefly, CO cells plated overnight on microscopic cover glasses in six-well plates transfected with zebrafish TBK1 (2μg), or infected with SVCV or GCRV for 24h. After fixed with paraformaldehyde and permeabilized with Triton X-100, the cells were blocked with 10% ADB (10% goat serum、3% BSA, 0.05% Triton X-100) at 37°C for 1 h, incubated overnight with the first antibody of IRF3 and IRF7 at 4°C and then with Alexa Fluor Plus 555 TRITC (Invitrogen) fluorescent second antibody and DAPI in the dark at 37°C for 1 h, followed by confocal microscope observation. The antibodies specific to crucian carp IRF3 and IRF7 was made by immunizing white rabbit with prokaryotic expressed DBD domains of IRF3/7 as described previously (31, 35).

### DNA Pull-Down Assays and Western Blots

DNA pull-down assays were performed as described previously (28, 34, 35). Briefly, HEK293T cells inoculated overnight in 10cm petri dishes were transfected with plasmids expressing GFP-fused IRF. 24h later, the cells were harvested in HKMG buffer with protease inhibitor (Roche) and further lysed by ultrasonication. One-tenth of cell lysates were taken as input. Appropriate amounts of cell lysates were incubated at 4°C with 15 μl M-280 streptavidin Dynabeads (Invitrogen), and biotinylated promoter DNA probes (20ng). The cell lysates should contain excessive overexpressed-IRF proteins to ensure the completed binding to DNA at a constant amount. Another 24h later, the beads were washed with HKMG buffer five times, followed by western blotting analysis of promoter-bound IRF protein with the antibody specific to GFP (Dia-an Biological Technology, Wuhan, China). The results were the representative of at least three independent experiments.

### Statistical Analysis

Student’s t-test is applied for statistical analysis of the data derived from luciferase assays and RT-PCR assays.

## Results

### Differential Induction of Zebrafish IRF Family Genes by SVCV Infection

**Figure 1:**
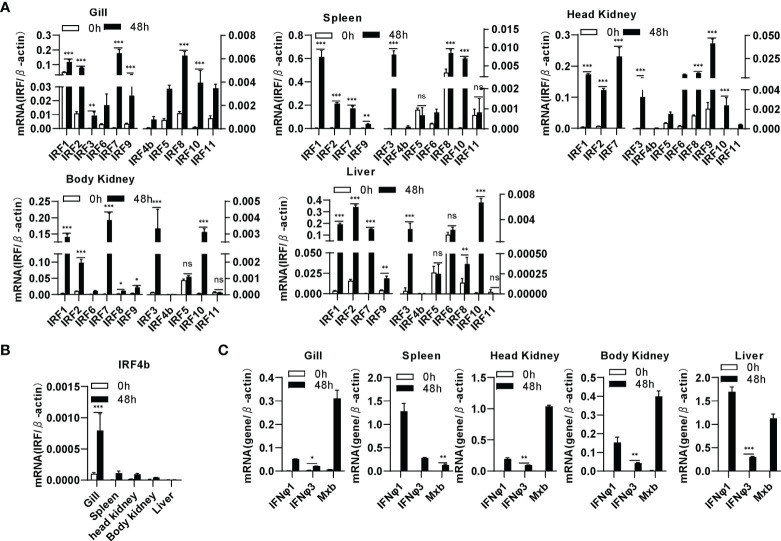
Zebrafish IRF family genes are transcriptionally induced in zebrafish tissues by SVCV infection. zebrafish adults were intraperitoneally injected with SVCV virus of 1×10^7^ TCID_50_/ml (25μL/fish). 48h later, five tissues, including gill, spleen, head kidney, body kidney, and liver, were sampled for extraction of total RNAs followed by qRT-PCR analyses of 11 zebrafish IRF family genes **(A)**, zebrafish IRF4b **(B)**, and zebrafish IFNφ1, IFNφ3 and Mxb **(C)**. The relative expression values of a given gene were normalized to β-actin in the same sample. Error bars represent SDs obtained by measuring each sample in triplicate. (***P < 0.001, **P < 0.01, *P < 0.05, ns, no significant).

By analyzing the genome data of zebrafish, we cloned a total of 11 IRF genes (IRF1-11) from zebrafish. There are two copies of IRF4 (IRF4a and IRF4b) reported in zebrafish genome (46). Zebrafish IRF4a is essential in T lymphoid-primed progenitors (47). In the study, IRF4b was cloned for further assays. Expression comparison showed that IRF1/2/3/7/8/9/10 were significantly upregulated by SVCV infection in all five zebrafish tissues (gill, spleen, head kidney, body kidney and liver), and IRF4b/5/6/11 did not to respond to viral infection in certain tissues, such as IRF6 in liver, IRF5 and IRF11 in spleen, body kidney and liver (**Figure 1A**). Of 11 zebrafish genes, IRF4b displayed the weakest induction in zebrafish tissues except for liver (**Figures 1A, B**). Under the same conditions, two zebrafish IFN (IFNφ1 and IFNφ3) and Mx genes were markedly induced (**Figure 1C**). These results indicated that zebrafish IRF family genes were differentially induced by SVCV infection when IFN response was activated.

### Differential Regulation of Zebrafish IRF Family Members in Fish IFN Response

**Figure 2:**
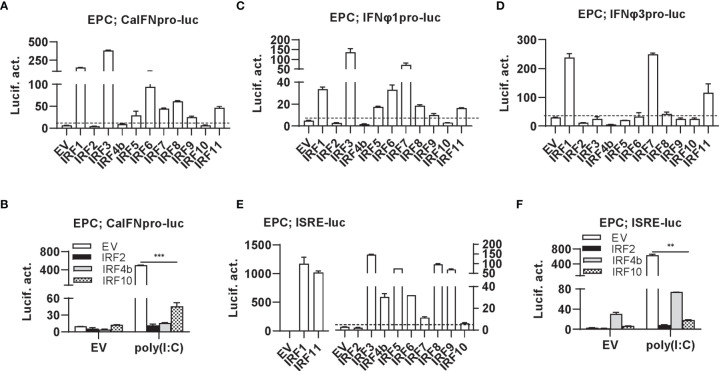
Zebrafish IRF proteins are capable to regulate fish IFN response. **(A)** overexpression of zebrafish IRF proteins regulate the activation of crucian carp IFN promoter. EPC cells seeded in 48-well plates overnight were transfected with CaIFNpro-luc (100 ng), each of zebrafish IRF plasmids (100 ng) and pRL-TK (10 ng). The control cells were transfected with pEGFP-N3 (EV) instead of IRF plasmid. 30h later, cells were harvested for luciferase assays. **(B)** overexpression of zebrafish IRF2/4b/10 inhibits poly(I:C)-triggered activation of crucian carp IFN promoter. EPC cells seeded in 48-well plates overnight were transfected with CaIFNpro-luc (100 ng), each of zebrafish IRF2/4b/10 (100 ng), pRL-TK (10 ng) and poly(I:C) (1 ug/ml) for 30h, followed by luciferase assays. **(C–E)** overexpression of zebrafish IRF proteins regulate the activation of zebrafish IFNφ1 promoter **(C)**, IFNφ3 promoter **(D)**, and ISRE-containing promoter **(E)**. EPC cells were transfected as in **(A)** with IFNφ1pro-luc **(C)**, IFNφ3pro-luc **(D)**, or ISRE-luc **(E)**. **(F)** overexpression of zebrafish IRF2/4b/10 inhibits poly(I:C)-triggered activation of ISRE-containing promoter. EPC cells were transfected as in **(B)** with ISRE-luc instead of CaIFNpro-luc. The dashed line indicates the basic stimulatory effect of empty vector on IFN promoter activation. The data shown were representative of three independent experiments. (***P < 0.001, **P < 0.01).

Luciferase assays revealed that overexpression of IRF1/3/5/6/7/8/9/11 significantly stimulated crucian carp IFN promoter-driven luciferase activity (CaIFNpro-luc), albeit to different degrees (**Figure 2A**). Instead, overexpression of IRF2/4b/10 did not show any stimulation (**Figure 2A**) but resulted in a decreased luciferase activity triggered by poly(I:C) transfection (**Figure 2B**). Zebrafish has four IFN genes, which are divided into group I and group II subfamilies, being represented by IFNφ1 and IFNφ3 (32). Crucian carp IFN and zebrafish IFNφ1 belong to group I IFN subfamily (48). Therefore, when IFNφ1pro-luc was used to replace CaIFNpro-luc, overexpression of IRF1/3/5/6/7/8/9/11 or IRF2/4b/10 yielded similar results (**Figure 2C**). Compared to IFNφ1pro-luc, IFNφ3pro-luc was activated only by IRF1/7/11, but not by IRF3/5/6/8 (**Figure 2D**), indicating that zebrafish IFNφ1 and IFNφ3 are transcriptionally regulated by different IRF family members.

Similarly, the IFN activators IRF1/3/5/6/7/8/9/11 were capable to activate ISRE-containing promoters (ISRE-luc) (**Figure 2E**), and IRF2/4b/10 downregulated poly(I:C)-triggered activity of ISRE-luc (**Figure 2F**). Notably, despite inability to stimulate fish IFN promoters (**Figure 2A**), overexpression of IRF4b alone yielded 10-fold luciferase activity of ISRE-luc over the control (**Figures 2E, F**). These results together indicate that zebrafish IRF members have the ability to regulate the expression of IFN genes and ISGs, with some (IRF1/3/5/6/7/8/9/11) functioning as positive regulators of IFN gene transcription, and others (such as IRF2/4b/10) as negative regulators. In addition, IRF3/5/6/8 differentially activates different fish IFN gene expression.

### Differential Binding of Zebrafish IRF Family Members to Fish IFN Promoters

**Figure 3:**
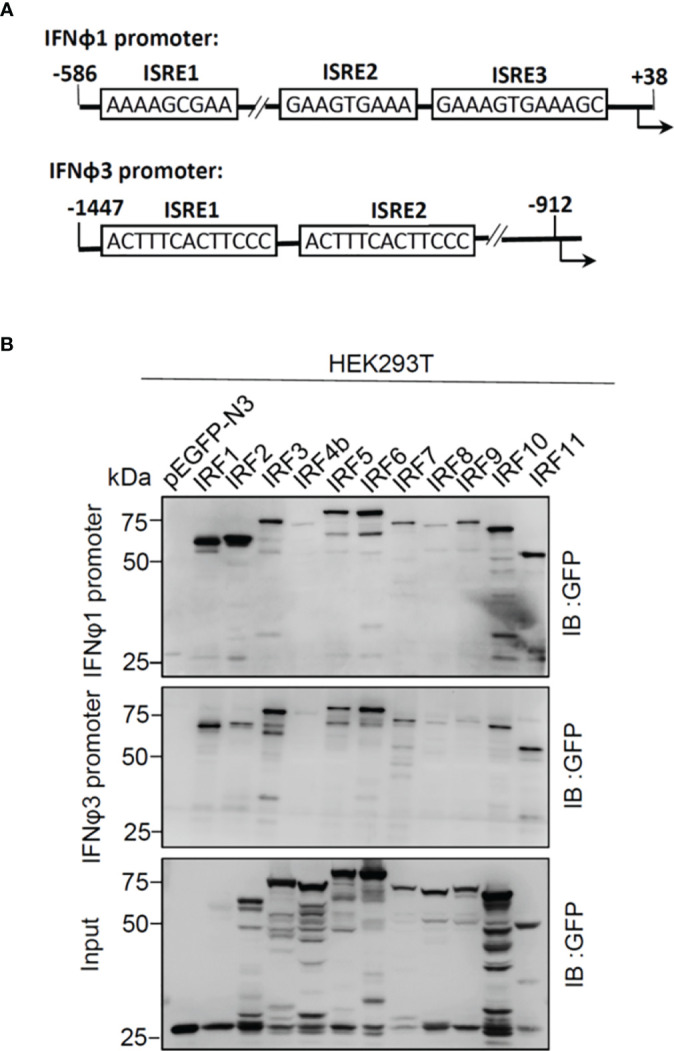
Zebrafish IRF proteins bind to zebrafish promoter DNA. **(A)** Schematic diagram of zebrafish IFNφ1 and IFNφ3 promoters, showing the position and sequence of potential ISRE/IRF-E motifs. **(B)** The binding of zebrafish IRF proteins to zebrafish IFN promoter DNA. The biotin-labeled DrIFNφ1 (-586 to +38) or DrIFNφ3 (-1447 to -910) promoter DNA (20 ng each) was incubated with the excessive amounts of zebrafish IRF proteins, or with GFP as control. Zebrafish IRF and GFP proteins were derived from the lysates of HEK293T cells that had been transfected for 30h with plasmids expressing zebrafish IRF protein fused to GFP or pEGFP-N3, respectively. One-tenth of cell lysates were taken as input. The bead-bound DNA-protein complex was detected by the Ab specific to the GFP tag using Western blotting.

There are three ISRE/IRF-E motifs within IFNφ1 promoter and two in IFNφ3 promoter (**Figure 3A**), which are the binding sites of DBDs of crucian carp IRF3 and zebrafish IRF1/3/7/11 (28, 34, 35). We wondered whether this might be the case for the other zebrafish IFN proteins. To this end, DNA pull-down assays were performed by incubation of biotin-labeled promoter DNAs of IFNφ1 (-586 to +38) or IFNφ3 (-1447 to -912), with IRF proteins that were overexpressed in HEK293T cells. Western blot analysis of promoter-bound products showed that all zebrafish IRF proteins effectively bound to IFNφ1/3 promoters (**Figure 3B**). Of 11 zebrafish IFN proteins, IRF4b and IRF8 displayed the weakest binding to two fish promoter DNA, followed by IRF9.

### Differential Subcellular Localization of Zebrafish IRF Family Members

**Figure 4:**
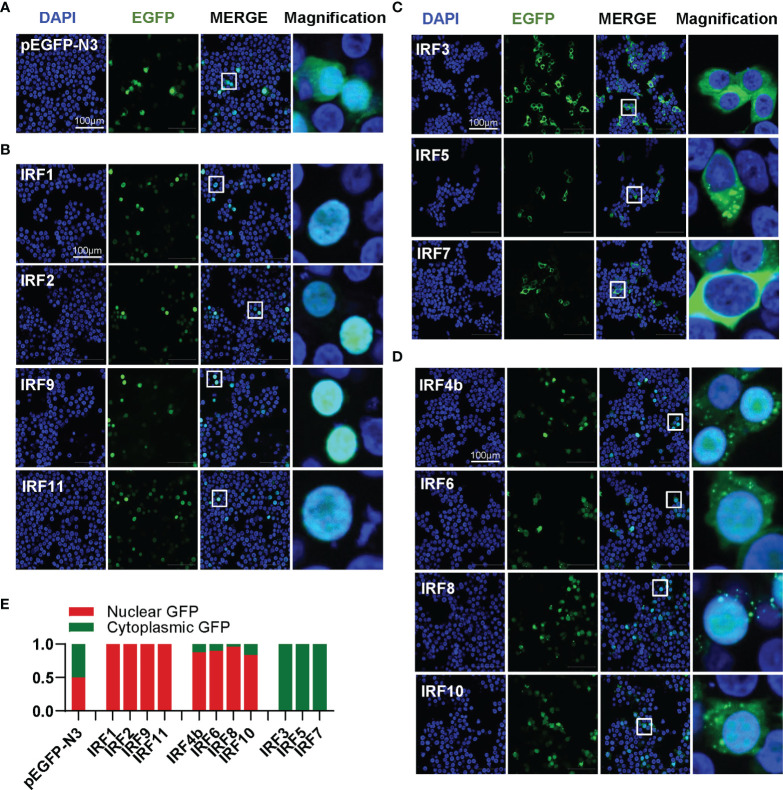
Zebrafish IRF family members show three patterns of constitutively subcellular localization. **(A–D)** Confocal microscopy illuminates the subcellular localization of zebrafish IRF proteins. HEK293T cells seeded on microscope slide cover glasses overnight in six-well plates were transiently transfected with pEGFP-N3 as control **(A)**, or with each of GFP-fused IRF1/2/9/11 constructs **(B)**, or with each of GFP-fused IRF3/5/7 constructs **(C)**, or with each of GFP-fused IRF4b/6/8/10 (2μg each). At 24 h post transfection, cells were fixed and examined using a confocal microscopy. DAPI staining showed the nuclei. The last column showed magnification view of the area highlighted in the box. **(E)** The intensities of nucleus/cytoplasm GFP were quantitated by the ImageJ processing program followed by normalization to that of pEGFP-N3, which was set to 1:1.

Transcription factors have to translocate to nucleus first for their function; therefore, it is of great interest to investigate the subcellular localization of zebrafish IRF family proteins. To this end, the ORFs of IRF genes were fused to GFP for transfection of HEK293T cell. Confocal microscopy examination showed that, distinct from the control GFP protein that was expressed ubiquitously in the whole cells (**Figure 4A**), 11 zebrafish IRF proteins exhibited three patterns of subcellular localization (**Figures 4B–D**). The first is nuclear accumulation, including IRF1/2/9/11 (**Figure 4B**). The second is cytoplasmic accumulation, including IRF3/5/7 (**Figure 4C**). And the third contains IRF4b/6/8/10 accumulating largely in nucleus and partially in cytoplasm (**Figure 4D**). Quantitation of nuclear/cytoplasmic GFP intensities supported the conclusions above (**Figure 4E**).

### K78 and R82 of α3 Helices in DBD Domain of IRF11 Are Conserved Across IRF Family Members

**Figure 5:**
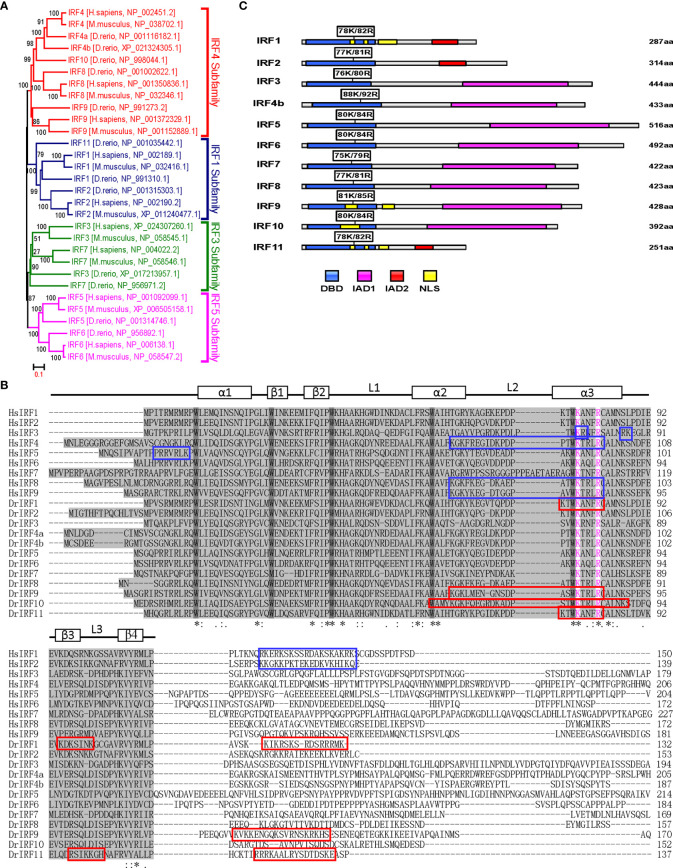
K78 and R82 of α3 helices in DBD domain of IRF11 are conserved across IRF family members. **(A)** Phylogenetic tree analysis of zebrafish and human IRF family members showing four IRF subfamilies. The phylogenetic tree was constructed by a neighbor-joining method in MEGA 5.0. The bootstrap confidence values shown at the nodes are based on 1000 bootstrap replications. **(B)** Multiple alignments of zebrafish IRF proteins showing a highly conserved DBD and the distribution of NLS motifs identified previously. DBD domains are gray with five conserved tryptophans. The symbols, including α-helices, β-strands and loops, indicates the secondary structures of DBD, which are marked by rectangle and line. Based on publications, the identified NLS motifs of zebrafish IRF1/10/11 are indicated by red boxes, and the NLS motifs of human IRF1/2/3/4/5/8/9 in blue boxes. Zebrafish IRF11 has a tripartite NLS motif composed of NLS1, NLS2 and NLS3, with the conserved K78 and R82 that are highlighted in purple. Identical (*) and similar (: or.) amino acid residues are indicated. **(C)** Schematic diagram of zebrafish IRF proteins, showing the position of two conserved basic residues corresponding to K78 and R82 of zebrafish IRF11. All mutants of zebrafish IRF protein were generated by combined mutation of the corresponding two residues to alanine.

Similar to previous results (28, 30) zebrafish IRF family can be divided into four subfamilies: IRF1 subfamily (IRF1/2/11), IRF3 subfamily (IRF3/7), IRF4 subfamily (IRF4/8/9/10) and IRF5 subfamily (IRF5/6) (**Figure 5A**). Multiple alignments showed zebrafish IRF proteins displays a high level of homology in DBDs and a weak one in C-terminal IAD) (**Figure 5B**). Based on human IRF1 structure (5), the DBDs should form a conserved α/β architecture, with a cluster of three α-helices (α1–α3) flanked on one side by a mixed four-stranded β-sheets (β1–β4) (**Figure 5B**).

We recently revealed that zebrafish IRF11 constitutively localizes to nucleus, exclusively dependent on a function-integrated tripartite NLS motif, which is composed of 3 aa stretches: NLS1 (aa75-82) and NLS2 (aa97–103) within DBD, and NLS3 (aa119–133) immediately adjacent to the C-terminal DBD (28) (**Figure 5B**). Notably, the regions corresponding to IRF11 NLS1, particularly two basic residues K78 and R82, are conserved across zebrafish and human IRF family, and instead, the regions corresponding to the NLS2 and NLS3 are less conserved (**Figure 5B**). Since K78 and R82 of zebrafish IRF11 contribute to its DNA-binding and nuclear import (28), we hypothesized that both amino acids have similar roles in other IRF family proteins. To this end, we generated IRF mutants by collective mutation of both basic residues to alanine (**Figure 5C**), to further characterize the DNA binding and nuclear retention features of zebrafish IRF family members.

### Differential Effects of Zebrafish IRF1/2/11 Mutants on IFN Response

**Figure 6:**
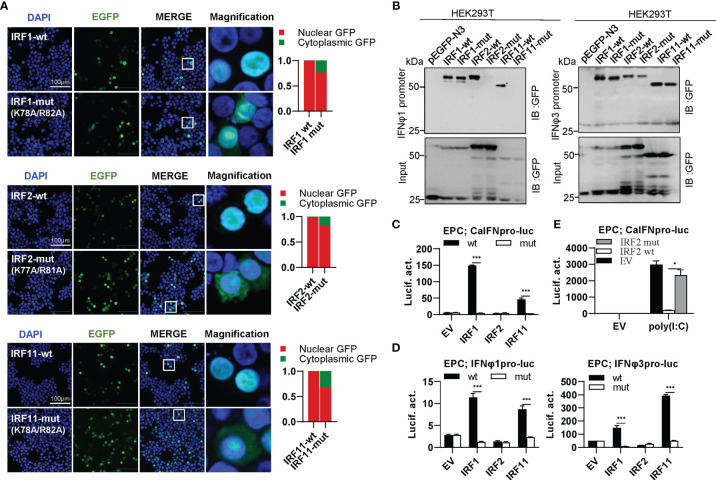
The two basic residues are essential for IRF1/2/11 to regulate IFN response. **(A)** Mutation of the two basic residues impaired the constitutively nuclear accumulation of IRF1/2/11. Left panels: HEK293T cells seeded overnight on microscope slide cover-glasses in six-well plates were transiently transfected as in **Figure 4**, with indicated IRF plasmids (2μg each) for 24h, followed by confocal microscopy examination. The last column showed magnification view of the area highlighted in the box. Right panels: The intensities of nucleus/cytoplasm GFP were quantitated using the ImageJ processing program and normalized to that of the empty construct pEGFP-N3, which was set to 1:1. **(B)** Pull-down analysis of the binding affinity of IRF1/2/11 wild types and IRF1/2/11 mutants to IFNφ1/IFNφ3 promoters. DNA pull-down assays were performed as in **Figure 3B**, by incubating biotin-labeled IFNφ1 or IFNφ3 promoter DNAs with appropriate amounts of HEK293T cell lysates, where cells were transfected for 30h with wild types or mutants of IRF1/2/11, respectively. **(C, D)** IRF1/11 mutants failed to stimulate the activation of crucian carp IFN promoter **(C)**, and zebrafish IFNφ1/IFNφ3 promoters **(D)**. EPC cells seeded in 48-well plates overnight were transfected with CaIFNpro-luc **(C)**, IFNφ1pro-luc or IFNφ3pro-luc **(D)**, together with the indicated IRF plasmids (100ng each) for 30 h, followed by luciferase assays. **(E)** IRF2 mutant failed to inhibit poly(I:C)-triggered activation of crucian carp IFN promoter by luciferase assays. EPC cells were transfected with the indicated plasmids (100ng each), together with poly(I:C) (1μg/ml) for 30h. (***P < 0.001, *P < 0.05).

IRF1 subfamily is composed of IRF1, IRF2 and IRF11. Compared to wild type IRF1/2/11 that exclusively localized to nucleus, collective mutation of both conserved amino acids to alanine (IRF1-mut, IRF2-mut and IRF11-mut) significantly altered their constitutive localization patterns, resulting in partially cytoplasmic localization (**Figure 6A**). Pull-down assays revealed that either IRF2-mut or IRF11-mut largely lost the binding affinity to DrIFNφ1 promoter DNA and slightly to DrIFNφ3 promoter DNA, but IRF1-mut appeared to have equal promoter binding affinity to the wild type IRF1 (IRF1-wt) (**Figure 6B**). Luciferase assays showed that, compared to the wild type IRFs, IRF1-mut and IRF11-mut failed to activate CaIFNpro-luc (**Figure 6C**), DrIFNφ1pro-luc and DrIFNφ3pro-luc (**Figure 6D**). Similarly, IRF2-mut completely lost the ability to downregulate the luciferase activity of CaIFNpro-luc triggered by poly(I:C) (**Figure 6E**). These results together indicated that mutation of both conserved amino acids of α3 helices in DBD domains resulted in a completely functional loss of three zebrafish IRF1 subfamily members, likely through impairing nuclear retention and DNA binding (IRF2 and IRF11), or only through impairing nuclear retention (IRF1).

### Differential Effects of Zebrafish IRF3/7 Mutants on IFN Response

**Figure 7:**
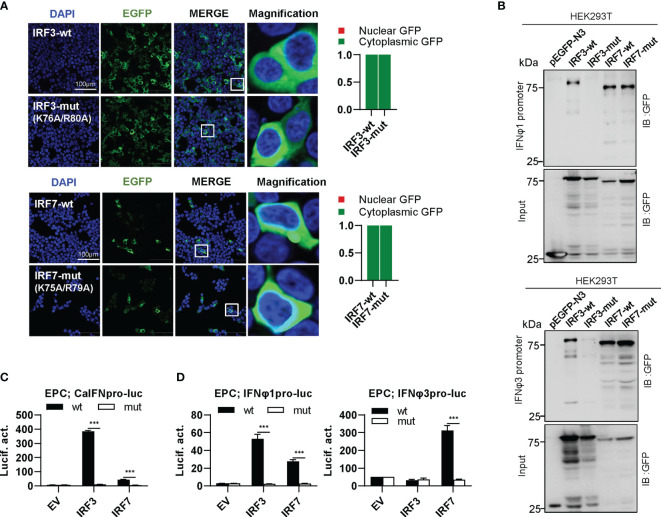
The two basic residues are essential for IRF3/7 to stimulate IFN response. **(A)** Mutation of two residues did not change the constitutively nuclear accumulation of IRF3/7. Left panels: HEK293T cells seeded overnight on microscope slide cover-glasses in six-well plates were transiently transfected as in **Figure 4**, with indicated plasmids (2μg each) for 24h, followed by confocal microscopy examination. The last column showed magnification view of the area highlighted in the box. Right panels: The intensities of nucleus/cytoplasm GFP were quantitated using the ImageJ processing program and normalized to that of the empty construct pEGFP-N3, which was set to 1:1. **(B)** Pull-down analysis of the binding affinity of wild types and mutants of IRF3/7 to IFNφ1/IFNφ3 promoters. DNA pull-down assays were performed as in **Figure 3B**, by incubation of biotin-labeled IFNφ1 or IFNφ3 promoter DNA with appropriate amounts of HEK293T cell lysates, where cells were transfected for 30h with wild types or mutants of IRF3/7, respectively. **(C, D)** IRF3/7 mutants failed to stimulate the activation of crucian carp IFN promoter **(C)**, and zebrafish IFNφ1/IFNφ3 promoters **(D)**. EPC cells seeded in 48-well plates overnight were transfected with CaIFNpro-luc **(C)**, IFNφ1pro-luc or IFNφ3pro-luc **(D)**, together with the indicated IRF plasmids (100ng each). 30 h later, cells were harvested for luciferase assays. (***P < 0.001).

IRF3 subfamily contains IRF3 and IRF7. In contrast to IRF1 subfamily members, wild type IRF3 and IRF7 (IRF3-wt and IRF7-wt) constitutively resided in cytoplasm (**Figure 7A**). Interestingly, combined mutation of both basic residues did not make any influence in constitutively cytoplasmic retention of IRF3 and IRF7 (**Figure 7A**), because both residues are essential for nuclear import but not nuclear export of zebrafish IRF11 (28). Compared to IRF3-wt and IRF7-wt that efficiently bound to both fish IFN promoters, IRF3-mut completely lost the DNA binding affinity, but no alteration was seen for IRF7-mut (**Figure 7B**). IRF3-wt mainly regulates the expression of group I IFN (crucian carp IFN and zebrafish IFNφ1) but not group II IFN (zebrafish IFNφ3) (**Figures 7C, D**). IRF7-wt has a strong stimulatory potential to zebrafish IFNφ3 and a weak one to zebrafish IFNφ1 (**Figure 7D**). Despite of these difference, combined mutation of both basic residues (IRF3-mut and IRF7-mut) completely abrogated their stimulatory potential to fish promoters (**Figures 7C, D**).

### Differential Effects of Zebrafish IRF5/6 Mutants on IFN Response

**Figure 8:**
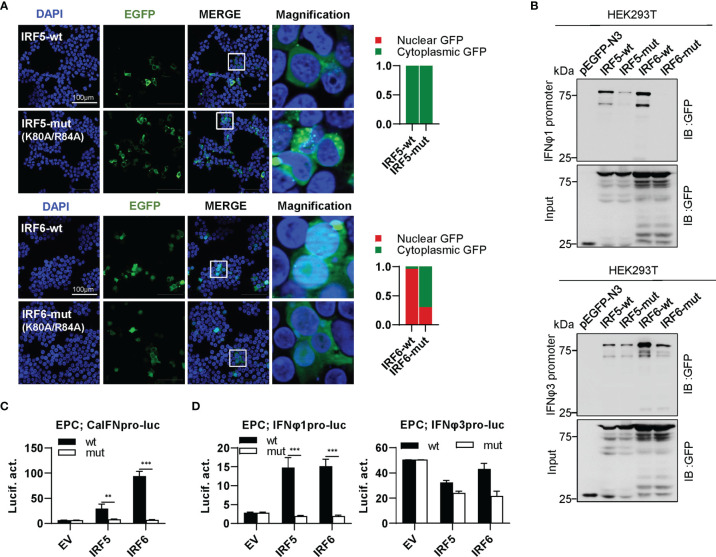
The two basic residues are essential for IRF5/6 to regulate IFN response. **(A)** Mutation of the two residues impaired the constitutively nuclear accumulation of IRF6 but not of IRF5. Left panels: HEK293T cells seeded overnight on microscope slide cover-glasses in six-well plates were transiently transfected as in **Figure 4**, with indicated plasmids (2μg each) for 24h, followed by confocal microscopy examination. The last column showed magnification view of the area highlighted in the box. Right panels: The intensities of nucleus/cytoplasm GFP were quantitated using the ImageJ processing program and normalized to that of the empty construct pEGFP-N3, which was set to 1:1. **(B)** Pull-down analysis of the binding affinity of wild types and mutants of IRF5/6 to IFNφ1/IFNφ3 promoters. DNA pull-down assays were performed as in **Figure 3B**, by incubating biotin-labeled IFNφ1 or IFNφ3 promoter DNA with appropriate amounts of HEK293T cell lysates, where cells were transfected for 30h with wild types or mutants of IRF5/6, respectively. **(C, D)**. IRF5/6 mutants failed to stimulate the activation of crucian carp IFN promoter **(C)**, and zebrafish IFNφ1/IFNφ3 promoters **(D)**. EPC cells seeded in 48-well plates overnight were transfected with CaIFNpro-luc **(C)**, IFNφ1pro-luc or IFNφ3pro-luc **(D)**, together with the indicated IRF plasmids (100ng each). 30h later, cells were harvested for luciferase assays. (***P < 0.001; **P < 0.01).

IRF5 subfamily is composed of IRF5 and IRF6. IRF5-wt was constitutively expressed in cytoplasm, and the same is true for IRF5-mut (**Figure 8A**, upper panel). Distinct from IRF5-wt, IRF6-wt largely accumulated in nucleus and slightly in cytoplasm; on the contrary, IRF6-mut was largely expressed in cytoplasm and partially in nucleus (**Figure 8A**, lower panel). These results indicated that mutation of both conserved amino acids have made a significant influence on the constitutively subcellular localization of IRF6 but not of IRF5. Regarding the promoter binding activity, IRF5-mut and IRF6-mut showed a weak or no binding to IFNφ1 promoter DNA, and a medium one to IFNφ3 promoter DNA compared to their wild types (**Figure 8B**). Despite that IRF5-wt and IRF6-wt effectively activated crucian carp IFN promoter, overexpression of IRF5-mut and IRF6-mut gave a basal luciferase activity, comparable to the control (**Figure 8C**). IRF5-wt and IRF6-wt selectively activated zebrafish IFNφ1 promoter but not zebrafish IFNφ3 promoter; similarly, IRF5-mut and IRF6-mut completely lost the ability to stimulate IFNφ1 promoters (**Figure 8D**).

### Differential Effects of Zebrafish IRF4b/8/9/10 Mutants on IFN Response

**Figure 9:**
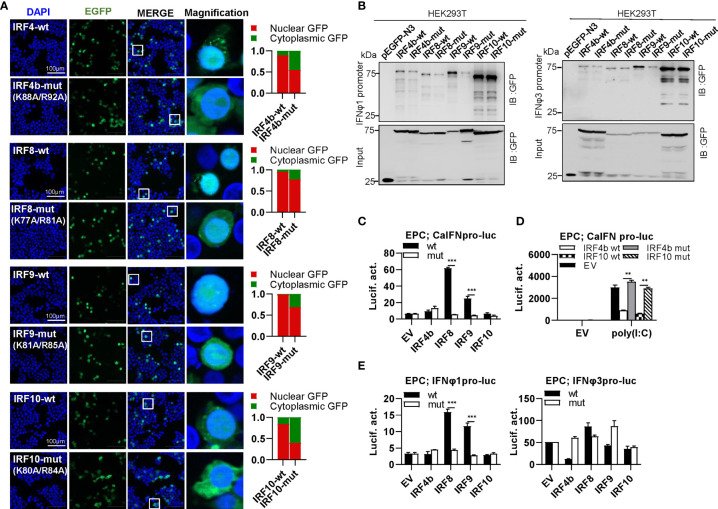
The two basic residues are essential for IRF4b/8/9/10 to regulate IFN response. **(A)** Mutation of the two basic residues impaired the constitutively nuclear accumulation of IRF4b/8/9/10. Left panels: HEK293T cells seeded overnight on microscope slide cover-glasses in six-well plates were transiently transfected as in **Figure 4**, with indicated plasmids (2μg each) for 24h, followed by confocal microscopy examination. The last column showed magnification view of the area highlighted in the box. Right panels: The intensities of nucleus/cytoplasm GFP were quantitated using the ImageJ processing program and normalized to that of the empty construct pEGFP-N3, which was set to 1:1. **(B)** Pull-down analysis of the binding affinity of wild types and mutants of IRF4b/8/9/10 to IFNφ1/IFNφ3 promoters. DNA pull-down assays were performed as in **Figure 3B**, by incubating biotin-labeled IFNφ1 or IFNφ3 promoter DNAs with appropriate amounts of HEK293T cell lysates, where cells were transfected for 30h with wild types or mutants of IRF4b/8/9/10, respectively. **(C)** IRF8/9 mutants failed to stimulate the activation of crucian carp IFN promoter. EPC cells seeded in 48-well plates overnight were transfected for 30h with CaIFNpro-luc, together with the indicated IRF plasmids (100ng each). **(D)** IRF4b/10 mutant failed to inhibit poly(I:C)-triggered activation of crucian carp IFN promoter. EPC cells were transfected with the indicated plasmids (100ng each), together with poly(I:C) (1μg/ml) for 30h. **(E)** IRF8/9 mutants failed to stimulate the activation of zebrafish IFNφ1/IFNφ3 promoters. EPC cells were transfected with IFNφ1pro-luc or IFNφ3pro-luc, together with the indicated IRF plasmids (100ng each). 30 h later, cells were harvested for luciferase assays. (***P < 0.001, **P < 0.01).

IRF4 subfamily consists of IRF4, IRF8, IRF9 and IRF10, showing two distinct patterns of constitutively subcellular localization, only in nucleus (IRF9), and most in nucleus with a little in cytoplasm (IRF4b/8/10) (**Figure 9A**). Regardless of which localization pattern they possessed, mutation of both basic residues significantly changed their constitutive localization, with much more proteins residing in cytoplasm (**Figure 9A**). Similar to mammalian counterparts (12, 49), zebrafish IRF4b and IRF8 exhibited a weaker binding to both zebrafish promoters than IRF9 and IRF10 (**Figure 9B**).Mutation of both amino acids in IRF4b/8/9 (IRF4b-mut, IRF8-mut and IRF9-mut) but not in IRF10 (IRF10-mut) severely impaired their binding to zebrafish IFNφ1 promoter, but no significant change was seen to zebrafish IFNφ3 promoter except for IRF9-mut (**Figure 9B**). Compared to the wild types, IRF8-mut and IRF9-mut did not activate crucian carp IFN promoter anymore (**Figure 9C**), and IRF4b-mut and IRF10-mut failed to downregulate poly(I:C)-triggered IFN promoter activation (**Figure 9D**). IRF8-wt and IRF9-wt obviously activated zebrafish IFNφ1 promoter but not zebrafish IFNφ3 promoter; consistently, IRF8-mut and IRF9-mut completely lost their immune-stimulatory potential (**Figure 9E**).

### Impaired Nuclear Import of Zebrafish IRF3/5/7 Mutants by Stimulation

**Figure 10:**
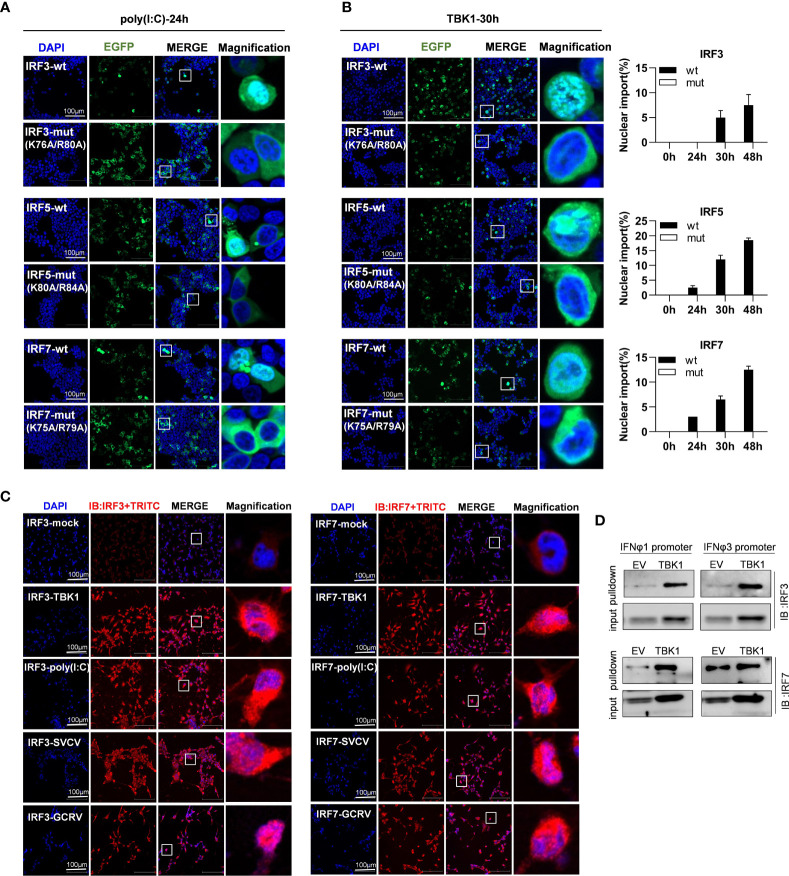
The two basic residues are essential for inducible nuclear import of IRF3/5/7 by transfection of poly(I:C) **(A)** and TBK1 **(B)**. **(A)** Mutation of the two basic residues impaired the inducible nuclear import of IRF3/5/7 by poly(I:C) transfection. HEK293T cells seeded overnight on microscope slide cover-glasses in six-well plates were transiently cotransfected as in **Figure 4** for 24h, with the indicated IRF plasmids (2μg each) and poly(I:C) (100ng/ml). **(B)** Mutation of the two basic residues impaired the nuclear import of IRF3/5/7 by overexpression of TBK1. HEK293T cells seeded overnight on microscope slide cover-glasses were transiently transfected as in **Figure 4**, with the indicated IRF plasmids and TBK1 (2μg each) for different time points (24, 30, 48h), followed by a time-course confocal microscopy examination. Left panels showed the representative images at 30h post transfection. Right panels: The ratio of the nuclear-translocated cells to total fluorescent cells was statistically quantitated by cell counting of the whole visual field under confocal microscopy. **(C)** Immunofluorescence microscopy observation of endogenous IRF3 and IRF7 proteins in cells with or without stimulation by IFN stimuli. CO cells seeded overnight on microscope slide cover-glasses were transiently transfected with TBK1 (2 μg) or poly(I:C) (100ng/ml or infected wit SVCV or GCRV(1×10^3^ TCID_50_/ml each), or mock treatment as control. 24h later, the cells were fixed, incubated overnight with polyclonal antibodies of fish IRF3 and IRF7, stained with fluorescent secondary antibody (Alexa Fluor Plus 555 TRITC). Red signal indicated endogenous IRF3 and IRF7 proteins. An enlarged view of the highlighted area in the last column display box. **(D)** Pull-down analysis of the binding affinity of Endogenous IRF3 and IRF7 proteins to fish promoter DNA. CO cells seeded overnight in 10cm^2^ dishes were transfected with TBK1 or empty vector (5μg each) for 30h. The biotin-labeled DrIFNφ1 (-586 to +38) or DrIFNφ3 (-1447 to -910) promoter DNA (30 ng each) was incubated with the transfected cell lysates overnight at 4°C, followed by western blotting to detect IRF3 and IRF7 proteins by fish antibodies specific to IRF3 and IRF7. Note: the low binding intensities in empty vector-transfected CO cells might be due to the low level of constitutive expressed IRF3 and IRF7, which finally resulted in unsaturated protein binding to DNA in the pulldown assays.

As mentioned above, mutation of both basic residues did not change the constitutively cytoplasmic retention of zebrafish IRF3/7 (**Figure 7A**) and IRF5 (**Figure 9A**). We wondered whether mutation of both basic residues impaired the induced nuclear import of IRF3/5/7. As shown in **Figure 10A**, in response to 24h-poly(I:C) transfection, less than 1% overexpressed cells showed a subcellular translocation of IRF3/5/7-wt from cytoplasm to nucleus. However, similar nuclear import was not observed for IRF3/5/7-mut under the same condition (**Figure 10A**).

To corroborate the findings, we replicated the assays using TBK1 instead of poly(I:C), since TBK1 is the protein kinase specific to IRF3/5/7 activation (8, 31, 32). A time-course observation showed that overexpression of TBK1 facilitated the cytoplasmic to nuclear accumulation of IRF3/5/7-wt in a time-dependent manner, but not of IRF3/5/7-mut by statistic counting of cells that had undergone nuclear import (**Figure 10B**). When TBK1 was overexpressed together, the green fluorescence signal that was spatially homogenous in cytosol was generally condensed, and finally converged to the peripheral nuclear membrane; however, only wild types of IRF3/5/7 rather than the mutants were observed to translocate from cytoplasm to nucleus (**Figure 10B**). These results indicate that although mutation of both basic residues does not alter the cytoplasmic accumulation of IRF3/5/7 in resting cells, but significantly impairs the nuclear import of IRF3/5/7 following poly(I:C)- or TBK1-transfection.

The inducible nuclear import of endogenous IRF3 and IRF7 proteins were next determined in CO cells stimulated by TBK1 or poly(I:C) transfection, or by GCRV or SVCV transfection (**Figure 10C**). Using the polyclonal antibodies specific to fish IRF3 and IRF7, immunofluorescence microscopy showed that in resting cells (IRF3-mock and IRF7-mock), a red signal was weakly observed in cytoplasm, indicating that the constitutively expressed IRF3 and IRF7 proteins resided in cytoplasm. However, in TBK1-overexpressed cells (IRF3-TBK1, IRF7-TBK1), a strong red signal was highlighted, and some obviously located to nucleus, indicating that TBK1 induced the expression of endogenous IRF3 and IRF7 proteins, some of which in turn translocated from cytoplasm to nucleus. Similar results were seen in poly(I:C)-transfected cells (IRF3-poly(I:C), IRF7-poly(I:C)), SVCV-infected cells (IRF3-SVCV, IRF7-SVCV) and GCRV-infected cells (IRF3-GCRV, IRF7-GCRV) (**Figure 10C**). Pulldown assays showed that zebrafish IFNφ1 and IFNφ3 promoter DNA could bind to the endogenous IRF3 and IRF7 proteins derived from CO cells that were transfected with empty vector or with TBK1 (**Figure 10D**). It is noted that the low binding intensities in empty vector-transfected CO cells might be due to the low level of constitutive expressed IRF3 and IRF7, which finally resulted in unsaturated protein binding to DNA in the pulldown assays.

## Discussion

In the present study, we reveal a conserved mechanism for IRF proteins to nuclear import and DNA binding. Zebrafish IRF family members have *in vitro* ability to participate in IFN response, with IRF1/3/5/6/7/8/9/11 functioning as activators and IRF2/4b/10 as repressors. The direct evidence comes from the findings that all IRF proteins efficiently bind to zebrafish fish IFN promoters by DNA pull-down assays, and thus they exhibit stimulatory or inhibitory potential to IFN promoters by luciferase assays. It is believed that host IFN response is necessarily regulated at appropriate levels through cooperative interaction of distinct IRF members in a cell-type- and viral-specific manner (7–9). How IFN response is fine-tuned might be, at least partially, illuminated by the expression characteristics of IRF family members in response to viral infection. RT-PCR showed that most of zebrafish IRF family genes (IRF1/2/3/7/8/9/10) were transcriptionally induced in all zebrafish tissues by SVCV infection, and others (IRF4b/5/6/11) were in limited tissues, to varied degrees. The diversified expression patterns imply that zebrafish IRFs might be selectively activated to impart IFN signaling specificity to different viral sensors. For example, fish IRF3 and IRF7 participate in multiple signaling pathways triggered by TLRs (33), RLRs (32) and DNA sensors (such as cGAS) (50); on the contrary, fish IRF1 and IRF11 seem not to function in RLR signaling pathway (28, 34). We also found that zebrafish IRF family members have been diversified to target distinct zebrafish IFN gene expression (IFNφ1 by IRF1/3/5/6/7/8/9/11, and IFNφ3 by IRF1/7/11). Notably, zebrafish IRF9 is capable to activate fish IFN promoter, consistent with previous findings that fish IFN can upregulate itself expression through the Jak-Stat pathway (41, 48, 51).

Our results highlight the relevance of nuclear import of IRF proteins in regulating host IFN response. Actually, most zebrafish IRF proteins reside constitutively in nucleus (IRF1/2/9/11) or largely in nucleus (IRF4b/6/8/10), apart from zebrafish IRF3/5/7 accumulating constitutively in cytoplasm. IRF3/5/7 are best known for their crucial roles in promoting IFN-dependent innate response in mammals (8) and in fish (32). Our results showed that IRF3/5/7 are expressed in cytoplasm, generally in an inactive state like their mammalian counterparts (9), and after stimulation by poly(I:C) and TBK1 transfection or virus infection, they rapidly undergo the cytoplasmic to nuclear translocation. The induced nuclear import represents a precisely regulatory mode, meaning that it can easily guarantee the onset of IFN response in virally-infected cells. The constitutive nuclear retention of most zebrafish IRF proteins (IRF1/2/9/11, and IRF4b/6/8/10) might imply an alternative regulation mode of IFN antiviral response. A recent study has shown that constitutively nuclear-localized human IRF1 is essential for the basal transcription of antiviral genes required for the defense against multiple viral infection (52). Similarly, nuclear-resided IRF9 synergizes with STAT2 to control the basal expression of ISGs in normal murine macrophages (26). Despite that IRF8 is found to be partially localized in cytoplasm, the nuclear-localized IRF8 works with IRF3 to enable rapid IFN gene transcription in human blood monocytes following viral infection (7). Considering that IRF11 is unique to fish genome and resides constitutively in nucleus (28), it might be particularly beneficial for fish species to defense against aquatic virus.

IRF proteins translocate to the nucleus depending on the NLS that is specifically recognized by nuclear transport proteins, which transport the protein into the nucleus (27). A typical NLS is often composed of a stretch rich in lysines (K) and arginines (R) termed monopartite NLS, two stretches termed bipartite NLS, or three stretches termed tripartite NLS (22, 28). Characterization of fish IRF1/11’s NLSs have revealed an overlapping sequence (28, 34, 42), which is positioned at the helix α3 of DNA binding domains (DBDs) in fish IRF1 (34, 42), IRF9 (41), IRF10 (53) and IRF11 (28) (**Figure 5B**). Interestingly, this sequence is also overlapped with the identified NLS in human IRF1 and IRF2 (24), IRF3 (21, 29), IRF4/8/9 (22, 25). And within the overlapping sequences, two basic residues (corresponding to K78 and R82 in zebrafish IRF11) are conserved across IRF family members in fish and mammals (**Figure 5B**). Importantly, K78 and R82 enable IRF11 to have a NLS with the integrated function of nuclear import ability and DNA binding activity, which is essential for zebrafish IRF11 to constitutively reside in nucleus and bind to IFN promoter DNA for initiation of IFN gene expression (28).

The data in the current study further reveal a conserved mechanism that the two conserved basic residues exactly contribute to the nuclear import, either constitutively or induced, of all zebrafish IRF family proteins (summarized in **Table 2**). Mutation of the corresponding two amino acids directly impaired the constitutively nuclear accumulation of IRF1/2/4b/6/8/9/10/11 and completely blocked the inducible nuclear import of IRF3/5/7 by poly(I:C) and TBK1, thus abrogating the stimulatory potential of all zebrafish family members to IFN response. That is, all zebrafish IRF mutants have lost the intact function on fish IFN promoter activation, a stimulatory role for IRF1/3/5/6/7/8/9/11 and an inhibitory role for IRF2/4b/10. Additionally, our results suggested that both conserved basic residues are also indispensable for the DNA binding activity of zebrafish IRF family proteins except for IRF1/7/10, highlighting that both residues contribute to nuclear import and DNA binding in most zebrafish IRF family proteins. Combined with the fact that both basic residues are highly conserved across vertebrate IRF family (28), it is likely that there is a general rule for the integrated function of both residues in DNA binding and nuclear import of most vertebrate IRF members. However, we do not know why both residues are unrelated to the DNA binding ability of zebrafish IRF1/7/10. Exceptionally, the previously identified NLS of human IRF5 does not include the corresponding two residues (22, 54); however, the two residues indeed specify zebrafish IRF5 to nuclear retention under stimulation by poly(I:C) and TBK1 transfection (**Figure 10**). Therefore, it is deserved for further clarifying the roles of both residues in nuclear import and DNA binding of mammalian IRF family proteins.

**Table 2:** Impairment of zebrafish IRF mutants on promoter activation, nuclear import and DNA binding.

	Showing no impairment	Showing impairment
Stimulatory or inhibitory potential to IFN promoters		IFNφ1: all IFNφ3: IRF1/7/11
Nuclear import		Constitutive: IRF1/2/4b/6/8/9/10/11 Inducible: IRF3/5/7
Promoter binding	IFNφ1: IRF1/7/10 IFNφ3: IRF1/7/10, IRF4b/8	IFNφ1: IRF2/3/4b/5/6/8/9/11 IFNφ3: IRF2/3/5/6/9/11

In mammals, IRF4/6/8 are believed to locate largely in nucleus and partially in cytoplasm (6, 22, 25); however, contradictory results happen to fish. A study in seahorse (*Hippocampus abdominalis*) showed that IRF4/8 are constitutively expressed in nucleus and IRF6 in cytoplasm (44), but in grass carp (*Ctenopharyngodon idella*), IRF8 is reported to reside in both cytoplasm and nucleus (43). Unlike mammalian IRF4s (25), zebrafish IRF4 is previously believed to accumulate in nucleus (46), but in the present study, zebrafish IRF4b is mainly located in nucleus and partially in cytoplasm, which is consistent with mammalian IRF4s. These disparities might result from the usage of distinct experiment methods. For example, GFP fused to a given target protein was often used to illuminate nuclear localization (21, 22, 24, 25, 55); however, a marginal localization alteration is hard to discriminate from background, and thus is easily neglected by fluorescence microscopy. In the present study, we discriminate the exact localization signaling from background by cell counting of the whole visual field, finally quantifying and verifying that there are three different types of constitutively subcellular localization of zebrafish IRF members. Our results indicate the fact that mutation of the two basic amino acids seriously impair the constitutively or induced nuclear import of all zebrafish IRF members.

Overall, our results provide a comprehensive insight into the regulatory roles of zebrafish IRF family in IFN antiviral response. Two conserved basic residues within DBDs of zebrafish IRF family members are identified as a part of NLS, which directly contributes to nuclear import for all zebrafish IRF family members and DNA binding for most members, thereby enabling them to function as transcription factors of IFN response. Mutation of both residues abrogate the transactivation of all zebrafish IRF proteins, indicating a possibility that they might be essential in all vertebrate IRF family members because they are conserved in vertebrate IRF proteins. The fact that zebrafish IRF proteins constitutively reside in nucleus or cytoplasm also suggest that they mediate constitutive as well as inducible host IFN response toward viral infection.

## Data Availability Statement

The original contributions presented in the study are included in the article/supplementary files. Further inquiries can be directed to the corresponding author.

## Ethics Statement

The animal study was reviewed and approved by the Animal Care and Use Committee of Institute of Hydrobiology, Chinese Academy of Sciences.

## Author Contributions

LLA and YBZ designed the research. LLA, XZ, XYG, and YLL performed the experiments. YBZ, LLA, XYG, XZ, YLL, ZLQ, HYS, WHG, and CD analyzed the data. JFG provided useful insights and reagents. LLA and YBZ wrote the paper. All authors contributed to the article and approved the submitted version.

## Funding

This work was supported by the Grants from the Application Fundamental Frontier Special Project of Wuhan (2020020601012256), the Strategic Priority Research Program of the Chinese Academy of Sciences (XDA24010308), the National Key R&D Program of China (2018YFD0900302), and the National Natural Science Foundation (31772875 and 31972826).

## Conflict of Interest

The authors declare that the research was conducted in the absence of any commercial or financial relationships that could be construed as a potential conflict of interest.

## Publisher’s Note

All claims expressed in this article are solely those of the authors and do not necessarily represent those of their affiliated organizations, or those of the publisher, the editors and the reviewers. Any product that may be evaluated in this article, or claim that may be made by its manufacturer, is not guaranteed or endorsed by the publisher.

## Abbreviations

IRF, IFN regulatory factor; IRF-E, IRF-binding element; ISG, IFN-stimulated gene; ISRE, IFN-stimulated regulatory element; poly(I:C), polyinosinic:polycytidylic acid
